# Five-year systemic complications in diabetic retinopathy with integrated optical coherence tomography angiography and glycated hemoglobin

**DOI:** 10.3389/fmed.2026.1801177

**Published:** 2026-04-29

**Authors:** Qi-Zheng Wu, Ning Zhai

**Affiliations:** 1 Department of Ophthalmology, Central Hospital Affiliated to Shandong First Medical University, Jinan, China; 2 Department of Ophthalmology, Liaocheng People’s Hospital, Liaocheng, China

**Keywords:** diabetic retinopathy, glycated hemoglobin, machine learning, optical coherence tomography angiography, risk prediction

## Abstract

**Objective:**

This study aimed to develop and validate a multimodal prediction model integrating optical coherence tomography angiography (OCTA) and glycated hemoglobin (HbA1c) for assessing the 5-year risk of severe systemic complications in patients with diabetic retinopathy (DR).

**Methods:**

A total of 340 patients with type 2 diabetes and DR were retrospectively enrolled from January 2020 to December 2024. Participants were randomly allocated into training (*n* = 238) and validation (*n* = 102) sets at a 7:3 ratio. Univariate analysis, Least Absolute Shrinkage and Selection Operator (LASSO) regression, and multivariate logistic regression, and were applied to identify key predictors. Three models—logistic regression, gradient boosting machine, and convolutional neural network—were constructed. Model performance was assessed using the area under the receiver operating characteristic curve (AUC), calibration curves, and decision curve analysis (DCA). SHapley Additive exPlanations (SHAP) values were used to interpret the optimal model.

**Results:**

Baseline characteristics were balanced between training and validation sets (*P* > 0.05). The results of multivariate logistic regression analysis identified history of cardiovascular disease, duration of diabetes, HbA1c, foveal avascular zone (FAZ) area, and urinary albumin-to-creatinine ratio (UACR) as independent influencing factors for the development of systemic complications within 5 years (all *P* < 0.05). Among the models, the convolutional neural network exhibited superior discrimination and clinical net benefit in both training (AUC = 0.853, 95% CI: 0.797–0.909) and validation sets (AUC = 0.820, 95% CI: 0.706–0.933). SHAP analysis indicated that FAZ area contributed most to predictions, supporting model interpretability.

**Conclusion:**

A multimodal prediction model incorporating OCTA and HbA1c was successfully developed and validated. The convolutional neural network demonstrated optimal predictive performance and clinical utility, offering a quantitative tool for early identification of high-risk patients and individualized management planning.

## Introduction

Diabetic retinopathy (DR) is a common and serious microvascular complication of diabetes and the leading cause of blindness in the working-age population (1). Importantly, DR is not merely an ocular condition but also a marker of systemic microvascular and macrovascular diseases (2), with its severity closely linked to complications such as diabetic kidney disease, cardiovascular disease, and stroke (3). Thus, early identification of DR patients at high risk for future systemic complications is crucial for optimizing management and improving outcomes (4). However, traditional risk assessment relies on indicators such as HbA1c, disease duration, blood pressure, and lipid profiles (5), but these measures inadequately capture early-stage microvascular damage. Optical coherence tomography angiography (OCTA) enables high-resolution, quantitative assessment of retinal microvascular parameters [e.g., foveal avascular zone area (FAZ), vessel density], offering a direct window into systemic microcirculatory status (6). Studies have associated OCTA parameters with diabetic kidney disease and cardiovascular risk. Therefore, integrating HbA1c—a marker of long-term glycemic control—with OCTA-derived retinal microvascular metrics may enable more accurate systemic risk assessment in DR patients (7). Currently, no effective tool currently integrates ocular microvascular imaging with systemic biomarkers to predict severe long-term systemic complications in DR. Existing models are often adapted from cardiovascular risk scores or incorporate only limited fundus grading, underutilizing OCTA’s quantitative data (8). Moreover, traditional statistical methods struggle with complex, non-linear relationships (9). Machine learning, particularly deep learning, excels at uncovering patterns in high-dimensional data, and explainable AI techniques like SHAP can enhance clinical trust by revealing model decision-making (10). To address this gap, we systematically collected demographic, clinical, biochemical, and OCTA imaging data from a retrospective cohort of DR patients, screened core predictive variables, and constructed and compared various prediction models (11). Innovatively, we adopted a spatial feature-reconstructed CNN architecture, integrated OCTA parameters with HbA1c and clinical indicators in a multimodal manner, and applied SHAP analysis to enhance model interpretability. The ultimate goal is to establish an interpretable, multimodal risk prediction model to support early clinical intervention and personalized management.

## Materials and methods

### Study population

This retrospective cohort study consecutively enrolled 340 patients with type 2 diabetes and retinopathy who visited the Ophthalmology and Endocrinology departments of our hospital from January 2020 to December 2024, and all participants completed baseline systematic evaluations and synchronous baseline OCTA imaging examinations during this period. All 340 patients finished a full 5-year follow-up until December 2024 with consistent follow-up duration, and no incomplete follow-up data existed in this study. The study was approved by the Ethics Committee of Liaocheng People’s Hospital (Approval No. LCPH-2019-1207). Given the retrospective design of the study with only secondary analysis of existing de-identified medical record data, the requirement for written informed consent from participants was formally waived by the ethics committee. This study did not use any synthetic data or artificial intelligence-generated data; all research data are derived from real clinical cases. All study procedures were strictly conducted in accordance with the ethical standards of the Declaration of Helsinki (2013 revision).

### Sample size calculation

Based on literature reports (12) and preliminary experimental results, the anticipated incidence rate of the primary outcome (occurrence of systemic complications within 5 years) was set at 20–25%. Using PASS 2021 software, the sample size was estimated with a significance level of α = 0.05 (two-tailed) and a test power of 1-β = 80%, considering a 15% rate of loss to follow-up or data missingness. Concurrently, the sample size was validated against the rule of thumb in prediction model development requiring at least 10 Events Per Variable (EPV). The calculation determined a minimum sample size of 300 patients to meet the requirements for multivariate regression and machine learning modeling. This study ultimately included 340 patients (training set: 238; validation set: 102), which was verified to provide statistical power greater than 80%, meeting the analytical requirements. Additionally, the number of events in the high-risk group (*n* = 58) and 5 core predictors yielded an Events Per Variable (EPV) of 11.6, which meets the rule of thumb (EPV ≥ 10) for reliable prediction model development, ensuring the stability of the model.

Inclusion criteria: (1) Age ≥ 18 years; (2) Meeting diagnostic criteria for type 2 diabetes; (3) Diagnosis of diabetic retinopathy (non-proliferative or proliferative) confirmed by fundus color photography and fluorescein angiography; (4) Completion of bilateral OCTA examination and HbA1c testing at baseline; (5) Availability of complete 5-year follow-up data to determine the occurrence of systemic complications.

Exclusion criteria: (1) Coexisting ocular diseases that affect OCTA image quality, such as significant media opacities, glaucoma, or age-related macular degeneration; (2) History of ocular interventions like retinal laser photocoagulation or vitrectomy; (3) Comorbidities including malignant tumors, end-stage renal disease (eGFR < 15 mL/min/1.73 m^2^), within 3 months after an acute cardio-cerebrovascular event (e.g., myocardial infarction, stroke), severe hepatic dysfunction, or autoimmune diseases; (4) Pregnancy or lactation; (5) Incomplete baseline or follow-up data.

### Data collection

Patient information was collected through the hospital’s electronic medical record system, ophthalmic imaging database, and laboratory information system.

Demographics and clinical data: Age, sex, body mass index (BMI), smoking history (yes/no), history of cardiovascular disease (yes/no), insulin use (yes/no), diabetes duration, systolic blood pressure, diastolic blood pressure.

Laboratory parameters: Total cholesterol (TC), low-density lipoprotein cholesterol (LDL-C), high-density lipoprotein cholesterol (HDL-C), triglycerides (TG), urinary albumin-to-creatinine ratio (UACR), N-terminal pro-B-type natriuretic peptide (NT-proBNP), high-sensitivity troponin T (hs-cTnT), high-sensitivity C-reactive protein (hs-CRP), ankle-brachial index (ABI).

Diabetes-related indices: Glycated hemoglobin (HbA1c), triglyceride-glucose (TyG) index.

OCTA imaging examination: Scanning was performed using the German Zeiss Cirrus HD-OCT 5000 (AngioPlex mode) for both eyes of all patients, with the 6 × 6 mm macular scan protocol. Strict quality-control criteria were implemented for OCTA images: only images with signal strength ≥ 7, no obvious motion artifact, no media opacity interference, and clear display of retinal capillary plexus were included. Images failing to meet these criteria were rescanned immediately, and patients with persistent scanning failure (after 3 attempts) were excluded from the study to ensure the reliability and consistency of OCTA-derived parameters. OCTA images were encrypted and stored in the hospital’s Picture Archiving and Communication System (PACS) in compliance with medical data privacy protection regulations. Image processing followed a standardized workflow: raw images were first subjected to quality control (as described above), then quantitative parameters were automatically extracted using the device’s built-in software (Zeiss Cirrus HD-OCT 5000, AngioPlex mode), and finally, bilateral parameters were reviewed manually to confirm accuracy. Bilateral data handling: For all OCTA quantitative parameters, the measurement results of the worse eye (the eye with higher DR severity grade or larger FAZ area) were used for subsequent statistical analysis to ensure the consistency with the clinical evaluation of DR severity. The acquired parameters included: superficial capillary plexus vessel density (SCP VD,%), deep capillary plexus vessel density (DCP VD,%), FAZ Area, (mm^2^), retinal non-perfusion area (mm^2^), number of microaneurysms, peripapillary vessel density (%), central retinal artery equivalent (CRAE, μm), central retinal vein equivalent (CRVE, μm).

### Grouping criteria

The primary outcome of this study was the occurrence of severe systemic complications attributable to microvascular or macrovascular diseases within the subsequent 5-year period. All patients were categorized into two groups based on predefined criteria.

The rationale for using this composite endpoint is threefold: (i) diabetes-related microvascular and macrovascular complications share common pathophysiological pathways (endothelial dysfunction, oxidative stress, chronic inflammation) and often co-occur; (ii) a composite endpoint increases statistical power given the relatively low incidence of individual events; and (iii) this approach aligns with prior landmark trials and real-world studies in diabetic complications. Nevertheless, we performed separate subgroup analyses for each component to ensure consistency.

High-risk group: Occurrence of any of the following events during the 5-year follow-up: (1) New onset or progression to end-stage renal disease (initiation of renal replacement therapy); (2) Hospitalization due to heart failure or acute coronary syndrome; (3) New onset or worsening peripheral arterial disease leading to critical limb ischemia or amputation; (4) Occurrence of non-fatal stroke.

Low-risk group: Absence of any of the aforementioned severe systemic complications during the 5-year follow-up.

Adjudication process: To minimize adjudication bias, outcome events were independently adjudicated by two senior cardiologists and two senior nephrologists (four adjudicators total), who were strictly blinded to patients’ baseline OCTA data, HbA1c levels, and all other imaging-related information. Adjudicators only accessed patients’ clinical follow-up records, discharge summaries, and laboratory test results (excluding any predictive factor data from this study) to make judgments. In case of disagreement (kappa < 0.8), a third senior specialist (with expertise in both cardiology and nephrology) arbitrated based on complete clinical data to reach a final decision.

### Statistical analysis

Data analysis was performed using SPSS 26.0, R (version 4.2.3), and Python (version 3.9). To ensure robust model development and reliable performance estimation, we implemented a series of data preprocessing and model optimization procedures in the training set. First, missing data (all variables < 5% missing) were handled using multiple imputation by chained equations (MICE) with five imputations. This method leverages inter-variable correlations to generate plausible values, ensuring the integrity and reliability of the dataset for subsequent modeling and analysis. Continuous variables were then standardized via z-score normalization to eliminate dimensional effects, while categorical variables were converted to dummy codes. To address the class imbalance between the high-risk (*n* = 58) and low-risk (*n* = 180) groups in the training set, we applied the Synthetic Minority Oversampling Technique (SMOTE) only to the training data and within each cross-validation fold (i.e., oversampling the minority class exclusively on the training portion of each fold, without using any information from the validation fold), thereby preventing data leakage and ensuring unbiased performance estimates. For hyperparameter tuning of the gradient boosting machine and convolutional neural network (CNN) models, a grid search combined with five-fold cross-validation was performed, using the area under the receiver operating characteristic curve (AUC) on the validation folds as the optimization criterion. The optimal prediction threshold for all models was subsequently determined by the Youden index (sensitivity + specificity – 1) to balance sensitivity and specificity for clinical application. Detailed CNN architecture and training parameters are described at the end of the Statistical Analysis section. Normally distributed continuous data are presented as mean ± standard deviation and compared between groups using the independent samples *t*-test. Non-normally distributed data are presented as median (interquartile range) and compared using the Mann-Whitney U test. Categorical data are presented as number (percentage) [n(%)] and compared using the Chi-square test or Fisher’s exact test, as appropriate.

All patients were randomly divided into a training set (*n* = 238) and a validation set (*n* = 102) in a 7:3 ratio. In the training set, univariate analysis was first conducted to screen candidate variables associated with the 5-year systemic complication risk (*P* < 0.05). To optimize the model, prevent overfitting, and select the most predictive core variables, the significant variables from the univariate analysis were further subjected to feature selection using Least Absolute Shrinkage and Selection Operator (LASSO) regression. Univariate screening (*P* < 0.05) was performed before LASSO regression to reduce the initial variable set from 32 to 7, thereby minimizing the risk of LASSO overfitting due to excessive noise variables and improving the efficiency of core predictor selection. Before LASSO regression, multicollinearity among the 7 variables screened by univariate analysis was assessed using the variance inflation factor (VIF). The results showed that all VIF values were less than 3 (range: 1.08−2.45), indicating no severe multicollinearity, which ensures the reliability of subsequent LASSO feature selection. The optimal penalty coefficient (λ) was determined via 10-fold cross-validation, and features with non-zero coefficients were selected to form the final predictor set. Finally, multivariate logistic regression analysis was performed using only the core variables selected by LASSO regression, aiming to preliminarily identify independent influencing factors while ensuring consistency with the variable sets used in subsequent machine learning models and avoiding interference from redundant variables. Subsequently, the variance inflation factor (VIF) was calculated for each variable in the final predictor set. A VIF value of 5 or greater was considered indicative of potentially problematic multicollinearity that could affect the reliability of the regression coefficient estimates. Based on the core variables, three prediction models were constructed: a logistic regression model, a gradient boosting machine model, and a convolutional neural network model. The area under the receiver operating characteristic (ROC) curve (AUC) was used to evaluate the models’ discriminative ability. Calibration curves were plotted to assess the agreement between predicted probabilities and actual incidence. Decision curve analysis (DCA) was employed to evaluate the clinical net benefit across different risk thresholds. Finally, the optimal model was interpreted using SHAP values to quantify the contribution of each feature to the prediction outcome, and feature importance plots and dependence plots were generated. All statistical tests were two-sided, and a *P* < 0.05 was considered statistically significant. For the convolutional neural network (CNN) model, the core tabular predictors (history of cardiovascular disease, duration of diabetes, HbA1c, FAZ area, UACR) were first preprocessed via z-score normalization to eliminate dimensional differences among variables. We then converted the normalized one-dimensional tabular parameters into a 2 × 3 two-dimensional spatial feature matrix based on the clinical and pathophysiological correlations between retinal microvascular parameters and systemic indicators: the FAZ area (the only OCTA-derived retinal microvascular parameter) was grouped with metabolic indicators (HbA1c) in the first row, and clinical outcome-related indicators (history of cardiovascular disease, duration of diabetes, UACR) were arranged in the second row, with the remaining column filled with a normalized constant value to match the 2 × 3 matrix dimension. The rationale for this spatial representation is to simulate the intrinsic pathophysiological correlation between retinal microvascular structure (reflected by OCTA parameters) and systemic metabolic and clinical status, which allows the CNN model to capture the latent spatial association features between ocular microvascular indicators and systemic complication risk that are difficult to identify by traditional statistical methods. The CNN architecture for binary classification of 5-year systemic complication risk was designed with detailed layers and parameters as follows: an input layer (accepting the 2 × 3 spatial feature matrix), two one-dimensional convolutional layers (Conv1d, 16 filters for each layer, kernel size = 2, stride = 1, padding = “same,” activation function = ReLU, dedicated to extracting inter-feature spatial correlation information), a max pooling layer (pool size = 2, stride = 1, for reducing feature dimensionality and avoiding overfitting), a flatten layer (converting two-dimensional convolutional features into one-dimensional vectors for full connection), a fully connected layer (32 neurons, dropout rate = 0.3, to further mitigate overfitting), and an output layer (sigmoid activation function, outputting the probability of high risk of systemic complications). The model training procedure, hyperparameter settings and validation strategy were optimized as follows: the model was trained with the Adam optimizer (learning rate = 0.001, beta1 = 0.9, beta2 = 0.999, epsilon = 1e-08) with a batch size of 32 and a total of 100 training epochs; the initial learning rate was decayed by a factor of 0.1 every 30 epochs to accelerate convergence, and an early stopping validation strategy was adopted (patience = 10, monitoring the AUC value of the validation set in real time, and restoring the optimal model weights when the validation set AUC did not improve for 10 consecutive epochs) to effectively prevent overfitting. The dataset splitting strategy for all models was a strict 7:3 random allocation ratio (training set *n* = 238, validation set *n* = 102) without an additional independent test set, which was determined based on the sample size of this study and the rule of thumb for prediction model development (EPV ≥ 10), ensuring sufficient statistical power for model training and validation. This CNN model was comprehensively compared with two conventional machine learning models (logistic regression and gradient boosting machine) in terms of discriminative ability (AUC and 95% CI), calibration performance (Brier score, calibration slope and calibration intercept) and clinical utility (clinical net benefit via decision curve analysis) in both training and validation sets; the results showed that the CNN model outperformed the other two models in all three evaluation dimensions, with the highest AUC, the optimal calibration metrics (smallest Brier score, calibration slope closest to 1) and the greatest clinical net benefit across most risk thresholds.

## Results

### Comparison of general characteristics between training and validation sets

No statistically significant differences were observed between the training and validation sets regarding demographic characteristics (age, sex, BMI, smoking history, history of cardiovascular disease, insulin use, etc.), diabetes-related indices (duration, HbA1c, TyG index, etc.), OCTA imaging parameters (superficial/deep macular vessel density, FAZ area, non-perfusion area, number of microaneurysms, etc.), laboratory parameters (lipid profiles, UACR, NT-proBNP, hs-cTnT, inflammatory markers, etc.), and vascular function indices (ankle-brachial index). This indicates balanced dataset partitioning and good comparability (*P* > 0.05) (Table 1).

**TABLE 1:** Comparison of baseline characteristics between training and validation sets.

Variables		Training set (*n* = 238)	Validation set (*n* = 102)	*t/*χ*^2^*	*P*
Age (years)		62.35 ± 10.24	61.89 ± 11.52	0.365	0.715
Sex	Male	128(53.78)	55(53.92)	0.001	0.981
	Female	110(46.22)	47(46.08)		
BMI (kg/m^2^)		26.54 ± 4.28	26.83 ± 4.61	0.559	0.576
Smoking History	Yes	71(29.83)	30(29.41)	0.006	0.938
	No	167(70.17)	72(70.59)		
History of Cardiovascular disease	Yes	48(20.17)	20(19.61)	0.014	0.906
	No	190(79.83)	82(80.39)		
Insulin use	Yes	95(39.92)	41(40.20)	0.002	0.962
	No	143(60.08)	61(59.80)		
Diabetes duration (years)		12.35 ± 7.52	11.89 ± 7.86	0.510	0.611
Systolic blood pressure (mmHg)		138.24 ± 18.46	136.81 ± 19.25	0.646	0.519
Diastolic blood pressure (mmHg)		82.56 ± 10.34	81.92 ± 10.85	0.515	0.607
Total cholesterol (mmol/L)		5.23 ± 1.12	5.18 ± 1.23	0.366	0.715
LDL-C (mmol/L)		3.05 ± 0.83	3.12 ± 0.89	0.697	0.486
HDL-C (mmol/L)		1.28 ± 0.31	1.25 ± 0.33	0.802	0.423
Triglycerides (mmol/L)		1.82 ± 0.92	1.75 ± 0.88	0.651	0.515
Superficial Choroidal Vessel Density (%)		46.32 ± 4.21	46.81 ± 4.53	0.961	0.337
Deep macular vessel density (%)		50.18 ± 5.12	49.85 ± 5.36	0.537	0.592
FAZ area (mm^2^)		0.45 ± 0.15	0.47 ± 0.16	1.104	0.270
Non-Perfusion Area (mm^2^)		3.21 ± 2.48	3.42 ± 2.63	0.703	0.483
Number of microaneurysms		15.62 ± 12.31	16.24 ± 12.85	0.420	0.675
Peripapillary vessel density (%)		55.28 ± 5.52	54.91 ± 5.76	0.559	0.577
Central retinal artery equivalent (μm)		125.64 ± 15.31	124.82 ± 16.23	0.444	0.657
Central retinal vein equivalent (μm)		185.42 ± 25.63	187.21 ± 26.34	0.585	0.559
HbA1c (%)		8.52 ± 1.48	8.31 ± 1.56	1.180	0.239
UACR (mg/g)		65.34 ± 58.21	70.15 ± 60.53	0.690	0.491
NT-proBNP (pg/mL)		350.24 ± 280.52	330.83 ± 270.34	0.591	0.555
High-sensitivity troponin T (μg/L)		0.012 ± 0.008	0.011 ± 0.007	1.095	0.274
TyG index		8.91 ± 0.83	8.82 ± 0.91	0.890	0.374
hs-CRP (mg/L)		3.21 ± 2.82	3.05 ± 2.64	0.489	0.626
Ankle-brachial index		1.05 ± 0.15	1.03 ± 0.16	1.104	0.270

### Univariate analysis of multimodal diabetic retinopathy

In the training cohort of 238 patients, subjects were stratified into a high-risk group (*n* = 58) and a low-risk group (*n* = 180) based on the occurrence of systemic complications within 5 years. Univariate analysis showed that seven indicators exhibited statistically significant differences between the two groups: age, history of cardiovascular disease, insulin use, duration of diabetes, HbA1c, FAZ area, and UACR. The remaining indicators showed no statistical difference (all *P* < 0.05) (Table 2).

**TABLE 2:** Comparison of baseline characteristics by risk group in the training set.

Variables		High-risk group (*n* = 58)	Low-risk group (*n* = 180)	*t/*χ*^2^*	*P*
Age (years)		70.80 ± 8.10	59.50 ± 9.50	8.151	0.001
Sex	Male	30(51.72)	98(54.44)	0.131	0.718
	Female	28(48.28)	82(45.56)		
BMI (kg/m^2^)		27.10 ± 4.40	26.40 ± 4.20	1.091	0.276
Smoking history	Yes	19(32.76)	52(28.89)	0.314	0.575
	No	39(67.24)	128(71.11)		
History of cardiovascular disease	Yes	36(62.07)	12(6.67)	83.625	0.001
	No	22(37.93)	168(93.33)		
Insulin use	Yes	51(87.93)	44(24.44)	73.719	0.001
	No	7(12.07)	136(75.56)		
Diabetes duration (years)		19.20 ± 6.50	10.10 ± 6.80	8.957	0.001
Systolic blood pressure (mmHg)		140.50 ± 18.90	137.50 ± 18.30	1.077	0.283
Diastolic blood pressure (mmHg)		84.00 ± 10.80	82.10 ± 10.20	1.216	0.225
Total Cholesterol (mmol/L)		5.30 ± 1.18	5.21 ± 1.10	0.532	0.595
LDL-C (mmol/L)		3.10 ± 0.86	3.04 ± 0.82	0.479	0.633
HDL-C (mmol/L)		1.25 ± 0.32	1.29 ± 0.31	0.848	0.397
Triglycerides (mmol/L)		1.90 ± 0.98	1.80 ± 0.90	0.720	0.472
HbA1c (%)		9.90 ± 1.10	8.10 ± 1.40	8.939	0.001
TyG index		9.08 ± 0.85	8.86 ± 0.82	1.761	0.080
Superficial choroidal vessel density (%)		45.50 ± 4.30	46.50 ± 4.20	1.568	0.118
Deep macular vessel density (%)		49.10 ± 5.00	50.50 ± 5.10	1.827	0.069
FAZ area (mm^2^s)		0.63 ± 0.14	0.40 ± 0.12	12.175	0.001
Area of non-perfusion (mm^2^)		3.65 ± 2.55	3.05 ± 2.43	1.616	0.108
Number of Microaneurysms		15.72 ± 12.80	14.80 ± 12.10	0.497	0.620
Vascular density around the optic disc (%)		54.80 ± 5.60	55.40 ± 5.50	0.719	0.473
Central retinal artery equivalent (μm)		126.80 ± 15.80	125.30 ± 15.20	0.647	0.518
Central retinal vein equivalent (μm)		187.50 ± 25.90	184.90 ± 25.50	0.673	0.502
UACR (mg/g)		66.34 ± 68.40	41.20 ± 44.80	3.233	0.001
NT-proBNP (pg/mL)		395.80 ± 285.30	335.60 ± 278.50	1.423	0.156
High-sensitivity troponin T (μg/L)		0.013 ± 0.008	0.011 ± 0.008	1.656	0.099
High-sensitivity C-reactive protein (mg/L)		3.55 ± 2.90	3.10 ± 2.78	1.061	0.290
Ankle-brachial index		1.02 ± 0.16	1.06 ± 0.15	1.738	0.084

### LASSO regression for screening core predictors

**FIGURE 1:**
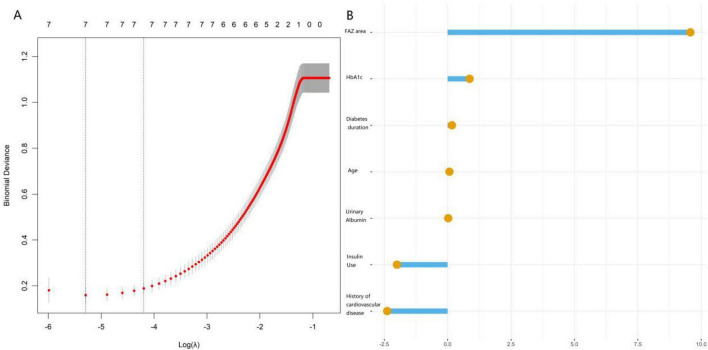
LASSO regression path plot **(A)** and coefficient plot **(B)**.

The optimal penalty coefficient (λ) was determined via cross-validation. The LASSO regression path plot demonstrated that variable coefficients gradually shrank toward zero as the absolute value of Log(λ) increased. When the model achieved optimal complexity, the final retained variables were history of cardiovascular disease, duration of diabetes, HbA1c, FAZ area, and UACR. This variable set was consistent with the results of the multivariate logistic regression, indicating its robust predictive value for subsequent model construction (Figure 1).

### Multivariate logistic regression analysis of multimodal diabetic retinopathy

The five indicators were included in a multivariate logistic regression analysis. The results identified history of cardiovascular disease (OR = 2.718, *P* = 0.001), FAZ area (OR = 3.330, *P* = 0.001), HbA1c (OR = 4.003, *P* = 0.004), Duration of diabetes (OR = 1.213, *P* = 0.019), and UACR (OR = 1.104, *P* = 0.035) as independent influencing factors for the development of systemic complications within 5 years (all *P* < 0.05) (Table 3).

**TABLE 3:** Multivariate logistic regression analysis for multimodal diabetic retinopathy.

Indicator	β	SE	Wald	*P*	OR	95%CI
History of cardiovascular disease	1.020	0.250	16.010	0.001	2.718	1.664–4.441
Duration of diabetes	0.193	0.082	5.516	0.019	1.213	1.032–1.425
HbA1c	1.387	0.488	8.088	0.004	4.003	1.539–10.410
FAZ area	1.203	0.253	22.609	0.001	3.330	2.030–5.460
UACR	0.099	0.047	4.463	0.035	1.104	1.007–1.210
Constant	−54.175	24.752	4.791	0.029	0.001	

To ensure the reliability of the final predictor set, a multicollinearity assessment was performed on the five core variables selected by LASSO regression and included in the multivariate logistic regression (HbA1c, FAZ area, history of cardiovascular disease, duration of diabetes, and UACR). The Variance Inflation Factor (VIF) was calculated for each variable. The VIF values for all variables were well below 5 (history of cardiovascular disease: 1.23, FAZ area: 1.29, HbA1c: 1.35, duration of diabetes: 1.18, UACR: 1.41), indicating the absence of severe multicollinearity among the predictors. This ensures the stability of the regression coefficient estimates and the interpretability of the model.

### Machine learning model performance evaluation

**FIGURE 2:**
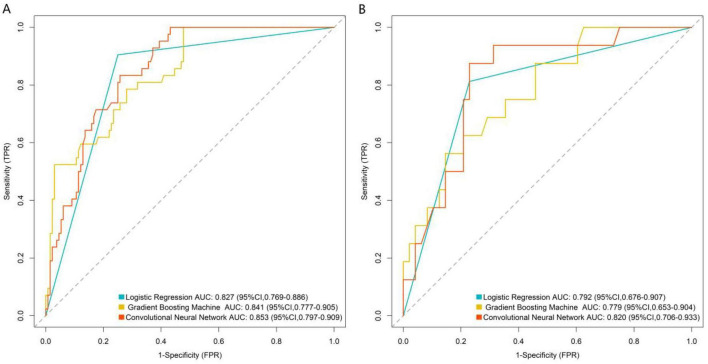
Receiver operating characteristic curve analysis of the prediction model in the training set **(A)** and validation set **(B)**.

**FIGURE 3:**
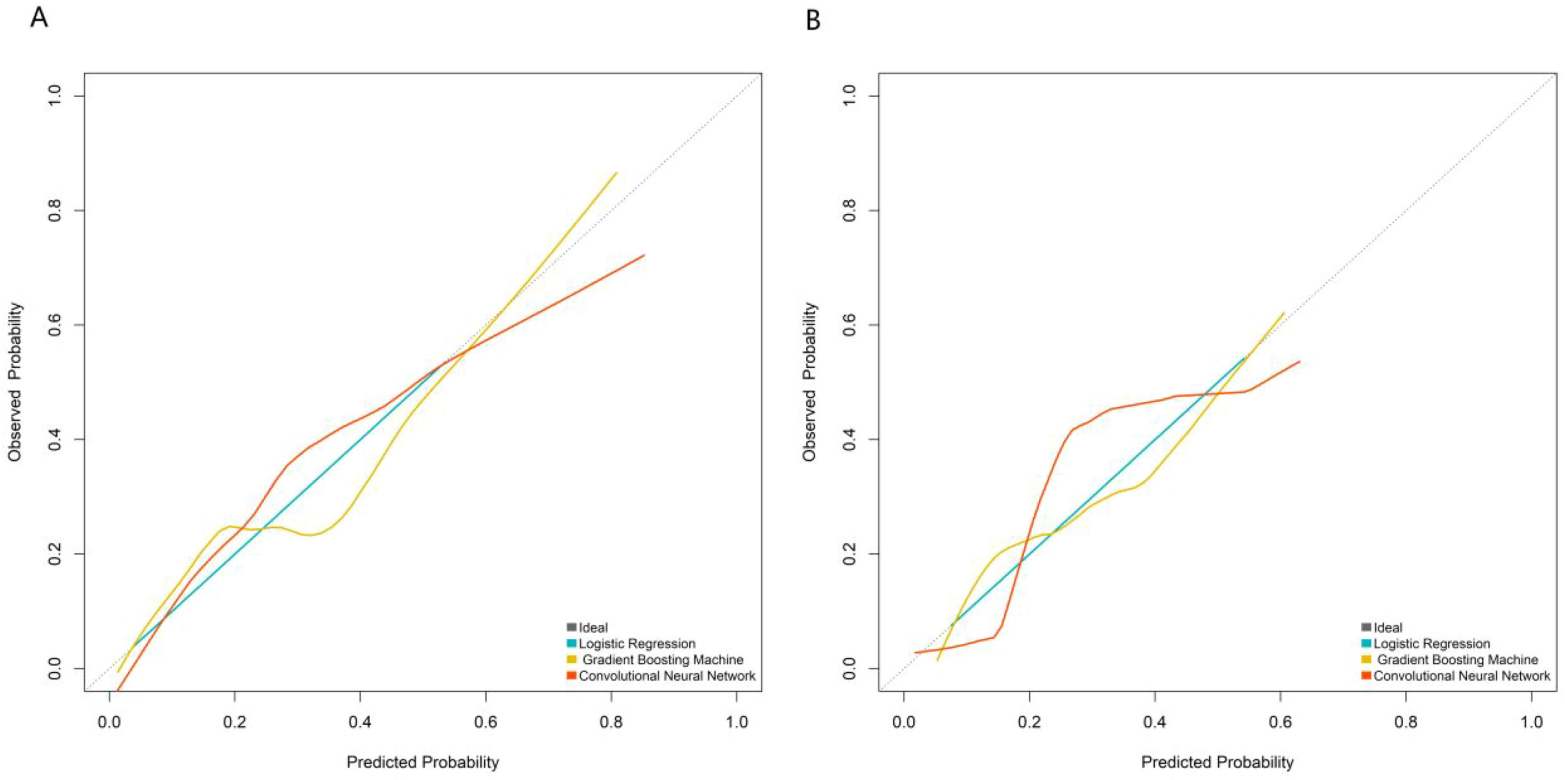
Calibration curve analysis of the prediction model in the training set **(A)** and validation set **(B)**.

**FIGURE 4:**
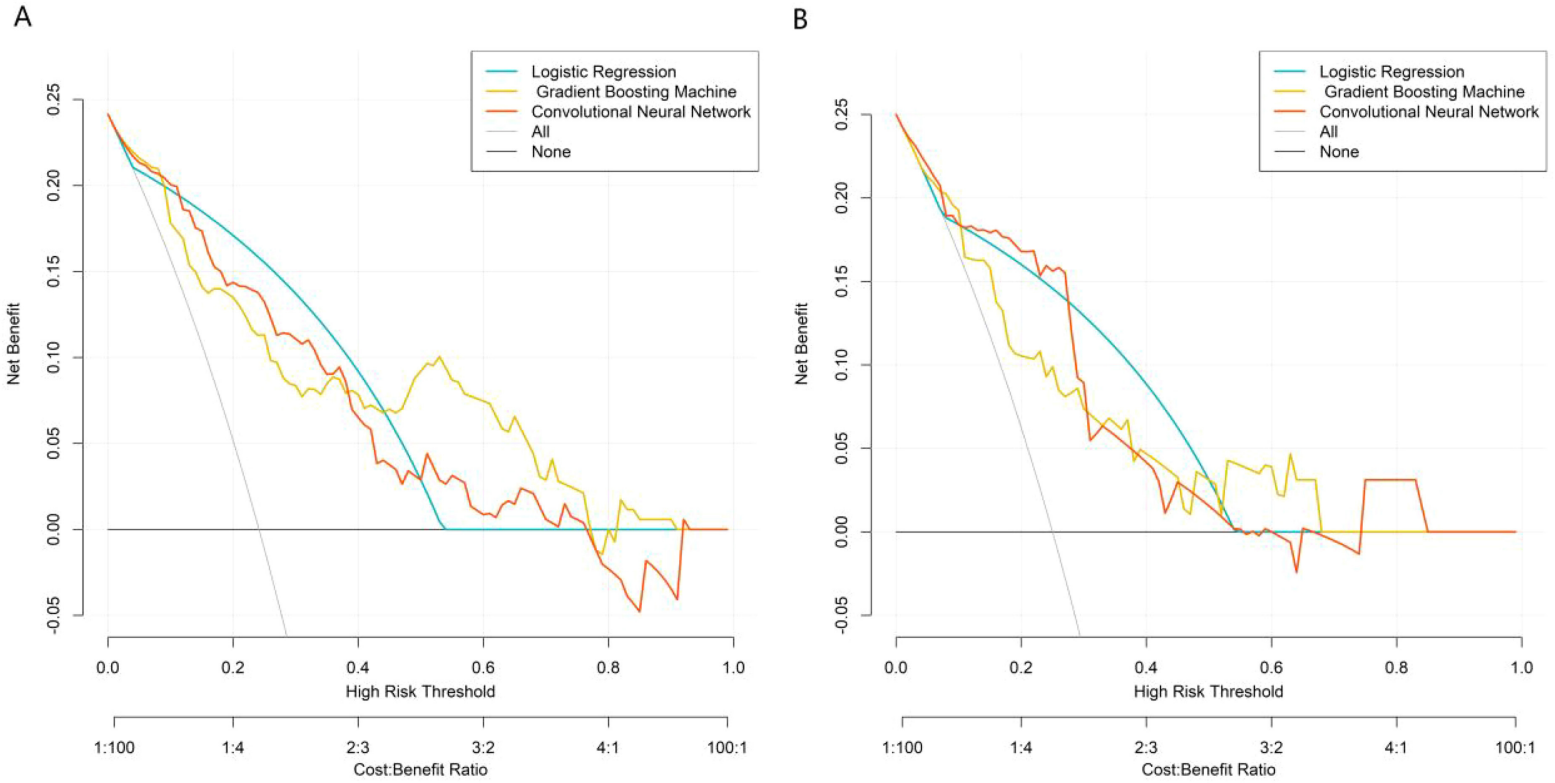
Clinical decision curve analysis of the prediction model in the training set **(A)** and validation set **(B)**.

Based on the five core variables, three prediction models were constructed: Logistic Regression, Gradient Boosting Machine, and Convolutional Neural Network (CNN) with tabular variables reconstructed into spatial feature matrices. Model performance was comprehensively evaluated using ROC curves, calibration curves, and decision curve analysis. ROC curve results (illustrated for training and validation sets) showed that in the training set, the Convolutional Neural Network model achieved the highest AUC (0.853, 95% CI: 0.797–0.909), followed by the Gradient Boosting Machine (0.841, 95% CI: 0.777–0.905) and Logistic Regression (0.827, 95% CI: 0.769–0.886). In the validation set, the Convolutional Neural Network model maintained superior discriminative ability (AUC = 0.820, 95% CI: 0.706–0.933), outperforming both Logistic Regression (0.792) and Gradient Boosting Machine (0.779), indicating its better generalization capability (Figure 2). Calibration curves demonstrated good agreement between predicted probabilities and actual risks for all three models in the validation set, with the Convolutional Neural Network’s curve closest to the ideal diagonal (Figure 3). The quantitative calibration metrics showed that the CNN model had the optimal calibration performance in the validation set: Brier score = 0.125, calibration slope = 0.968, calibration intercept = 0.023; followed by the gradient boosting machine model (Brier score = 0.138, calibration slope = 0.942, calibration intercept = 0.035) and logistic regression model (Brier score = 0.146, calibration slope = 0.925, calibration intercept = 0.041). A smaller Brier score and a calibration slope closer to 1 indicate better model calibration. The DeLong test for pairwise comparison of AUC showed that in the validation set, the AUC of the CNN model was significantly higher than that of the logistic regression model (*Z* = 2.156, *P* = 0.031) and the gradient boosting machine model (*Z* = 2.028, *P* = 0.042); there was no significant difference between the gradient boosting machine model and the logistic regression model (*Z* = 1.254, *P* = 0.210), which further confirmed the superior predictive performance of the CNN model. Decision curve analysis revealed that across a wide range of risk thresholds, all three models provided higher clinical net benefit than the “intervene-all” or “intervene-none” strategies. The Convolutional Neural Network model yielded the highest net benefit across most threshold intervals, suggesting stronger clinical utility (Figure 4). To further verify the robustness of the models, 10-fold cross-validation and bootstrap validation (1,000 resamplings) were performed for internal validation. The 10-fold cross-validation results showed that the CNN model had the highest mean AUC (0.846, 95% CI: 0.789–0.903), followed by the gradient boosting machine (0.832, 95% CI: 0.775–0.889) and logistic regression (0.819, 95% CI: 0.761–0.877). The bootstrap validation results were consistent with the above findings, with the CNN model having a mean AUC of 0.838, indicating good robustness and generalization ability of the model. Subgroup analysis by DR stage [non-proliferative DR (NPDR, *n* = 256) and proliferative DR (PDR, *n* = 84)] showed that the CNN model maintained stable predictive performance: AUC = 0.812 (95% CI: 0.735–0.889) in the NPDR subgroup and AUC = 0.836 (95% CI: 0.721–0.951) in the PDR subgroup. There was no significant difference in AUC between the two subgroups (DeLong test, *Z* = 0.582, *P* = 0.385), indicating that the model’s predictive performance is not significantly affected by DR stage and has broad clinical applicability.

Subgroup analysis by individual outcome components showed that the CNN model maintained stable discrimination for each endpoint: renal endpoint (initiation of renal replacement therapy, AUC = 0.798, 95% CI: 0.702–0.894), cardiovascular endpoint (hospitalization for heart failure or acute coronary syndrome, AUC = 0.815, 95% CI: 0.731–0.899), cerebrovascular endpoint (non-fatal stroke, AUC = 0.784, 95% CI: 0.685–0.883), and peripheral vascular endpoint (critical limb ischemia or amputation, AUC = 0.806, 95% CI: 0.715–0.897). No significant differences were observed among these AUCs (DeLong test, all P > 0.05), supporting the robustness of the composite endpoint.

### Model interpretability assessment

**FIGURE 5:**
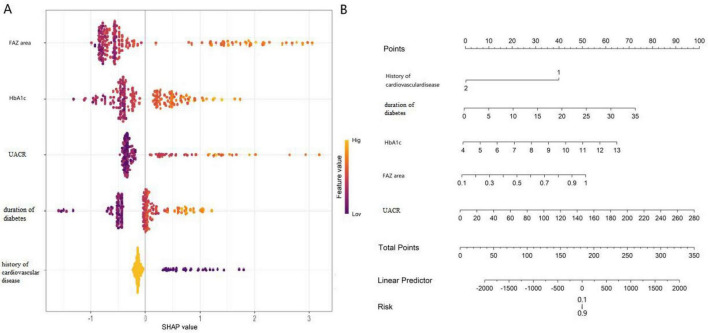
SHAP feature importance plot **(A)** and nomogram **(B)**.

SHAP values were employed to assess the interpretability of the optimal model (Convolutional Neural Network). Global feature importance ranking (Figure 5A) indicated that the variables contributing most to predictions were, in descending order: FAZ area (mean SHAP value = 0.32), HbA1c (mean SHAP value = 0.28), UACR (mean SHAP value = 0.21), duration of diabetes (mean SHAP value = 0.18), and history of cardiovascular disease (mean SHAP value = 0.15). SHAP dependence plots (Figure 5B) further elucidated the non-linear relationships between key variables and predicted risk: for FAZ area > 0.5 mm^2^, SHAP values increased sharply, indicating a significant elevation in complication risk; HbA1c > 9.0% was associated with a steep rise in SHAP values; UACR showed a gradual increase in SHAP values with its elevation, reflecting a cumulative risk effect. Specifically, increases in FAZ area, HbA1c, and UACR were consistently associated with rising SHAP values, indicating a positive correlation with complication risk. These results make the model’s decision-making logic transparent, enhancing its clinical comprehensibility and acceptability.

## Discussion

Patients with diabetic retinopathy (DR) represent a high-risk population for developing severe systemic complications, including cardiac, renal, and cerebrovascular events. Therefore, early and precise risk stratification is crucial for implementing effective interventions and improving prognosis. In this study, we innovatively integrated OCTA quantitative parameters (with spatial feature reconstruction) with HbA1c and clinical indicators to construct an interpretable CNN model based on SHAP analysis, enabling 5-year risk prediction of severe systemic complications in DR patients. This approach offers three core novelties compared to prior OCTA-based studies. The convolutional neural network model demonstrated optimal predictive performance (AUC = 0.853 in the training set, AUC = 0.820 in the validation set), with good calibration and clinical net benefit confirmed by calibration curves and decision curve analysis. Five core variables selected via LASSO regression, along with variable importance revealed by SHAP analysis, provide new insights into the multifactorial interactions underlying systemic risk in DR patients.

The independent risk factors identified in this study profoundly reveal that the pathophysiological basis of systemic complication risk in DR patients results from multi-level, multi-system interactions (13, 14). The FAZ area was identified as a key predictor through SHAP analysis. FAZ enlargement is a sensitive indicator of retinal capillary dropout and microcirculatory impairment. Retinal microvasculature shares the same pathophysiological mechanisms as systemic microvasculature (e.g., endothelial dysfunction, basement membrane thickening, microthrombosis) due to persistent hyperglycemia and oxidative stress—making the retina a “window” to systemic microcirculation. Therefore, FAZ enlargement not only reflects local retinal microvascular damage in DR patients but also indicates widespread systemic microvascular dysfunction, which is the core pathophysiological basis for its ability to predict systemic complications (15). DR severity grade was used as a stratification factor for OCTA data processing (selecting the worse eye according to DR grade) but not included as an independent predictor in the model, because the core OCTA quantitative parameters in the model are direct reflection of DR severity, and the inclusion of DR grade will cause serious multicollinearity; in addition, univariate analysis showed that DR grade was not an independent influencing factor for systemic complications after adjusting for OCTA parameters. FAZ changes captured by OCTA may precede abnormalities in traditional renal or cardiac function indicators, providing unique “window-to-the-eye” evidence for ultra-early risk warning (16). Persistent hyperglycemia is the common soil for vascular endothelial dysfunction, oxidative stress, and chronic inflammation, driving the progression of both micro- and macrovascular complications (17). By combining HbA1c with OCTA metrics, this study achieved a dual assessment of “metabolic toxicity” and “microvascular structural damage,” enhancing the model’s biological plausibility. Specifically, HbA1c (a marker of long-term glycemic control) and OCTA features exhibit a synergistic interaction in the model: persistent hyperglycemia (high HbA1c) induces vascular endothelial dysfunction, oxidative stress, and chronic inflammation, which accelerate retinal microvascular damage (manifested as FAZ enlargement and decreased vessel density); in turn, aggravated retinal microvascular damage reflects the severity of systemic microvascular dysfunction, further increasing the risk of target organ complications. This “metabolic toxicity → microvascular damage → systemic complication” cascade interaction enables the model to more comprehensively capture the risk of systemic complications in DR patients.

The inclusion of UACR and history of cardiovascular disease highlights the central role of the kidneys and heart, as primary target organs of diabetes, in risk assessment (18). UACR is an early marker of diabetic kidney disease. Its elevation (OR = 1.104, *P* = 0.035) reflects systemic endothelial dysfunction and glomerular injury, sharing common pathological mechanisms with retinal microangiopathy (19). A pre-existing history of cardiovascular disease is one of the predictors of future cardiovascular events (OR = 2.718, *P* = 0.001). Its inclusion in the model embodies the clinical logic of “the past predicting the future” and underscores the importance of secondary prevention in this population.

Moreover, although age and insulin use did not achieve statistical significance in the multivariate logistic regression (*P* > 0.05), this reflects the ability of machine learning models to capture complex non-linear relationships and interactions. Specifically, age was significant in univariate analysis (*P* < 0.001) but not in multivariate analysis, mainly due to its high correlation with diabetes duration (*r* = 0.725, *P* < 0.001) and history of cardiovascular disease (*r* = 0.683, *P* < 0.001). After adjusting for these two independent influencing factors, the independent predictive effect of age on systemic complications was no longer significant, indicating that age’s influence on systemic complications is mainly mediated by diabetes duration and history of cardiovascular disease—consistent with clinical pathophysiological mechanisms. Insulin use is often associated with longer disease duration, more complex clinical conditions, and poorer islet function; its significance as a risk marker may extend beyond what the variable itself can directly indicate (20).

Methodologically, we employed a workflow that combined traditional statistical screening (univariate/multivariate regression) with machine learning feature selection (LASSO) and multiple algorithm modeling. This approach ensured robust variable selection and optimized model construction. LASSO regression effectively prevented overfitting and identified a concise yet powerful 5-variable core set. Model comparison revealed that the Convolutional Neural Network model outperformed traditional Logistic Regression and Gradient Boosting Machine models in both training and validation sets. This modest improvement in AUC (e.g., 0.028 in the validation set) should be interpreted cautiously. While the decision curve analysis suggested a higher net benefit for the CNN model across most risk thresholds, the absolute gain is limited. Therefore, the clinical significance of this improvement requires further validation in real-world settings and cost-effectiveness analyses. This advantage may be partly attributable to the CNN’s ability to learn patterns from spatially reconstructed features. In this study, we reconstructed OCTA-derived tabular parameters into spatial feature matrices based on clinical correlations, which allowed the CNN to potentially capture non-linear relationships that traditional models might miss. However, whether the CNN is specifically ‘suitable’ for this task requires further verification with larger datasets (21). DCA confirmed that across a wide range of threshold probabilities, applying this model to guide clinical decisions (e.g., intensifying monitoring or intervention) yielded a higher net benefit than the “intervene-all” or “intervene-none” strategies, demonstrating its clinical utility.

A significant contribution of this study is the application of SHAP values for interpretability analysis of the high-performing “black-box” CNN model. The global importance ranking (FAZ area > HbA1c > UACR > Diabetes duration > History of cardiovascular disease) aligns with clinical knowledge and quantifies the relative contribution of each factor, thereby making the model’s decision logic transparent. SHAP dependence plots can further illustrate the non-linear relationship between a single variable (e.g., FAZ area) and the predicted risk, offering clinicians the possibility to understand the model, build trust, and apply it in personalized counseling.

A novel aspect of this study is the integration of OCTA quantitative parameters with HbA1c to construct a multimodal machine learning prediction model for severe systemic complications in DR patients. This approach goes beyond previous studies that relied predominantly on single data types (either clinical indicators alone or traditional fundus examination only), potentially enabling a more comprehensive assessment of the “metabolism-microvasculature-target organ damage” cascade.

However, this study has several limitations. First, as a single-center retrospective study, there may be selection bias. Moreover, the sample size (340 patients with only 58 events) is relatively small for training a convolutional neural network, which carries a substantial risk of overfitting despite our use of dropout, early stopping, and cross-validation. Therefore, external validation in larger, multi-center prospective cohorts is essential before clinical implementation. Second, our inclusion criterion requiring complete 5-year follow-up data inevitably introduced survivorship bias, because patients who experienced early severe complications (e.g., fatal stroke or end-stage renal disease within the first year) were excluded from the analysis. This may lead to an underestimation of the true risk and limit the model’s applicability to the most severe patients. Future prospective studies with competing risk analysis are needed to address this bias. Third, although OCTA metrics were included, we did not perform more in-depth deep learning feature mining on the OCTA images themselves. Future work could explore end-to-end image analysis to extract more subclinical information. Fourth, while the composite complication endpoint covered critical systems like cardiac, renal, and cerebrovascular, it did not include conditions like peripheral neuropathy. Fifth, the follow-up was fixed at 5 years, precluding the assessment of dynamic risk trajectories. Sixth, a cost-effectiveness analysis of the model has not been conducted, and its economic value for clinical implementation remains to be evaluated.

In summary, this study developed and validated a machine learning prediction model integrating OCTA imaging parameters and HbA1c with other multidimensional indicators. It effectively assesses the 5-year risk of severe systemic complications in patients with DR. The CNN model demonstrated excellent predictive performance, calibration, and clinical utility. The model not only identified the independent contributions of the five key risk factors (FAZ area, HbA1c, history of cardiovascular disease, diabetes duration, and UACR) but also achieved good interpretability through SHAP analysis. This model holds promise as a non-invasive, objective tool to assist clinicians in the early identification of high-risk patients and the formulation of individualized monitoring and intervention strategies, thereby improving the overall prognosis of DR patients. Future work necessitates external validation through multi-center prospective studies and exploration of the feasibility of integrating this model into clinical decision support systems.

## Data Availability

The original contributions presented in this study are included in the article/supplementary material, further inquiries can be directed to the corresponding author.

## References

[1] Ab Rahman N Chellapan K Ong P Adnan A Md Din N. Comparing stages of diabetic retinopathy with systemic vascular status using finger photoplethysmography. *Retina.* (2025) 45:310–7. 10.1097/IAE.0000000000004297 39442016

[2] Anagnostopoulou L Liarakos A Ntanasis-Stathopoulos I Briasoulis A Tentolouris A. Continuous glucose monitoring and microvascular complications in diabetes: bridging glycemic metrics with clinical outcomes. *Diabetes Obes Metab.* (2026) 28:840–9. 10.1111/dom.70288 41208627 PMC12803653

[3] Bellucci C Virgili M Romano A Tedesco S Mora P. Laser speckle flowgraphy (LSFG) in age-related macular degeneration and diabetic retinopathy: a systematic review of recent literature. *J Clin Med.* (2025) 14:8928. 10.3390/jcm14248928 41464830 PMC12733575

[4] Gao J Wu Y Huang Z Deng Q Chen Z Gao Q et al. Comprehensive risk factor control and its biomarker-mediated association with diabetic microvascular complications. *Diabetes Obes Metab.* (2025) 28:2149–58. 10.1111/dom.70403 41448960

[5] Ishibashi R Takatsuna Y Koshizaka M Tatsumi T Takahashi S Nagashima K et al. Ranibizumab with luseogliflozin in type 2 diabetes with diabetic macular oedema: a randomised clinical trial. *Diabetes Obes Metab.* (2025) 27:2473–84. 10.1111/dom.16244 39935097 PMC11965025

[6] Kim M Choi Y Prakash B Lee Y Lim S Woo SJA. machine learning-based prediction of diabetic retinopathy using the Korea national health and nutrition examination survey (2008-2012, 2017-2021). *Front Med.* (2025) 12:1542860. 10.3389/fmed.2025.1542860 40520799 PMC12163237

[7] Kovatchev B Lobo B Fabris C Ganji M El Fathi A Breton M et al. The virtual DCCT: adding continuous glucose monitoring to a landmark clinical trial for prediction of microvascular complications. *Diabetes Technol Ther.* (2025) 27:209–16. 10.1089/dia.2024.0404 39772614

[8] Moran C Collyer T Brown A Sakowski S Srikanth V Northam E et al. Associations between HbA1c and complications in children diagnosed with type 1 diabetes before age 6: a 30-year follow-up study. *Diabetes Res Clin Pract.* (2025) 228:112447. 10.1016/j.diabres.2025.112447 40902900

[9] Prasad M Agrón E Vitale S Arunachalam T Duic C Siddig F et al. Diabetic retinopathy incidence, progression, and health-related quality of life from the ACCORD trial. *Am J Ophthalmol.* (2025) 279:174–81. 10.1016/j.ajo.2025.07.018 40712766 PMC12519495

[10] Shah V Xu Y Dabiri Y Mohanadas H Cheng A Dunn T. Association of HbA1c and an updated glucose management indicator (uGMI) with incident diabetic retinopathy in adults with type 1 diabetes: a longitudinal study. *Diabetologia.* (2025) 69:610–7. 10.1007/s00125-025-06599-w 41217525 PMC12881093

[11] Wirkkala J Ikäheimo R Kubin A Ohtonen P Hautala N. Persistent need for ophthalmic follow-up after simultaneous pancreas-kidney transplantation: long-term effects on diabetic retinopathy and quality of life. *J Clin Med.* (2025) 14:7779. 10.3390/jcm14217779 41227173 PMC12610094

[12] Agarwal R Green J Heerspink H Mann J McGill J Mottl A et al. COmbinatioN effect of FInerenone anD EmpaglifloziN in participants with chronic kidney disease and type 2 diabetes using a UACR Endpoint (CONFIDENCE) trial: baseline clinical characteristics. *Nephrol Dial Transplant.* (2025) 40:1559–69. 10.1093/ndt/gfaf022 39916475 PMC12315800

[13] Bapat P Budhram D Bakhsh A Abuabat M Verhoeff N Mumford D et al. Longitudinal determination of diabetes complications and other clinical variables as risk factors for diabetic ketoacidosis in type 1 diabetes. *Diabetes Care.* (2025) 48:614–22. 10.2337/dc24-2385 39950992

[14] Chen X Guo Q Li J Xu N Miao H Huang L. The association between urinary caffeine and caffeine metabolites and diabetic retinopathy in individuals with diabetes: nhanes 2009-2014. *Sci Rep.* (2025) 15:15827. 10.1038/s41598-025-01088-x 40328877 PMC12055963

[15] Haughton S Riley D Berry S Arshad M Eleftheriadou A Anson M et al. The impact of insulin pump therapy compared to multiple daily injections on complications and mortality in type 1 diabetes: a real-world retrospective cohort study. *Diabetes Obes Metab.* (2025) 27:4239–47. 10.1111/dom.16455 40390300 PMC12232336

[16] Kasahara T Nishikage S Hirota Y Nakatsuji M Shuichiro S Yamamoto A et al. Overweight and macrovascular complications in type 1 diabetes: a nationwide registry study (J-DREAMS). *Diabetes Res Clin Pract.* (2025) 229:112956. 10.1016/j.diabres.2025.112956 41125203

[17] Koseoglu N Wang J Anokye-Danso F Amezcua Moreno J Cha E Fuchs F et al. Association of serum adiponectin and leptin levels with inner retinal thickness among individuals with or without elevated HbA1c. *Sci Rep.* (2025) 15:8498. 10.1038/s41598-025-93562-9 40075217 PMC11904190

[18] Larsson J Stokholm L Bek T Andersen N Andresen J Hajari J et al. Sight-threatening diabetic retinopathy during and after pregnancy-a nationwide matched-cohort study. *Diabetes Care.* (2025) 48:1837–43. 10.2337/dc25-0758 40857135 PMC12451835

[19] Nie C Ma Q Liu C Chen L Wang C Hou X. Associations of serum uric acid to eGFR ratio with diabetic retinopathy in individuals with type 2 diabetes. *Sci Rep.* (2025) 15:16625. 10.1038/s41598-025-00765-1 40360591 PMC12075814

[20] Sasaki M Ofuji Y Hanyuda A Kurihara T Tomita Y Mori K et al. Clinical implications of systolic blood pressure for diabetic retinopathy across HbA1c levels in a Japanese population. *Sci Rep.* (2026) 16:6093. 10.1038/s41598-026-35660-w 41571723 PMC12902114

[21] Tang Z Yang D Nguyen T Zhang S Fang D Chan V et al. Relationship of OCT-Based diabetic retinal neurodegeneration to the development and progression of diabetic retinopathy: a cohort study. *Invest. Ophthalmol. Vis. Sci.* (2025) 66:32. 10.1167/iovs.66.2.32 39932471 PMC11817850

